# 2954. Coordinated Antimicrobial Stewardship Efforts to Produce Sustained Reductions in Outpatient Fluoroquinolone Consumption

**DOI:** 10.1093/ofid/ofad500.193

**Published:** 2023-11-27

**Authors:** Corey M Frederick, Ernesto Sanz Martinez, Yenny Ceballos, Michael DeCoske, Philip Weimer, Richard Levine, Timothy P Gauthier

**Affiliations:** Baptist Health South Florida, Miami Lakes, Florida; Baptist Health South Florida, Miami Lakes, Florida; Baptist Health South Florida, Miami Lakes, Florida; Baptist Health South Florida, Miami Lakes, Florida; Baptist Health South Florida, Miami Lakes, Florida; Baptist Health South Florida, Miami Lakes, Florida; Baptist Health South Florida, Miami Lakes, Florida

## Abstract

**Background:**

Starting in 2020, antimicrobial stewardship efforts were coordinated to reduce outpatient consumption of FQs prescribed in 20 urgent care centers (UCCs) throughout a not-for-profit healthcare system. Coordinated efforts included clinician education on FQ treatment alternatives and direct prescriber audit and feedback in specific cases. Additionally, a shorter duration of a FQ regimen was introduced in automated pharmacy dispensing machines located throughout the UCCs. The study's objective was to assess if these coordinated efforts resulted in sustained reductions in FQ consumption several years after implementation.

Automated Pharmacy Dispensing Machine

**Figure:**
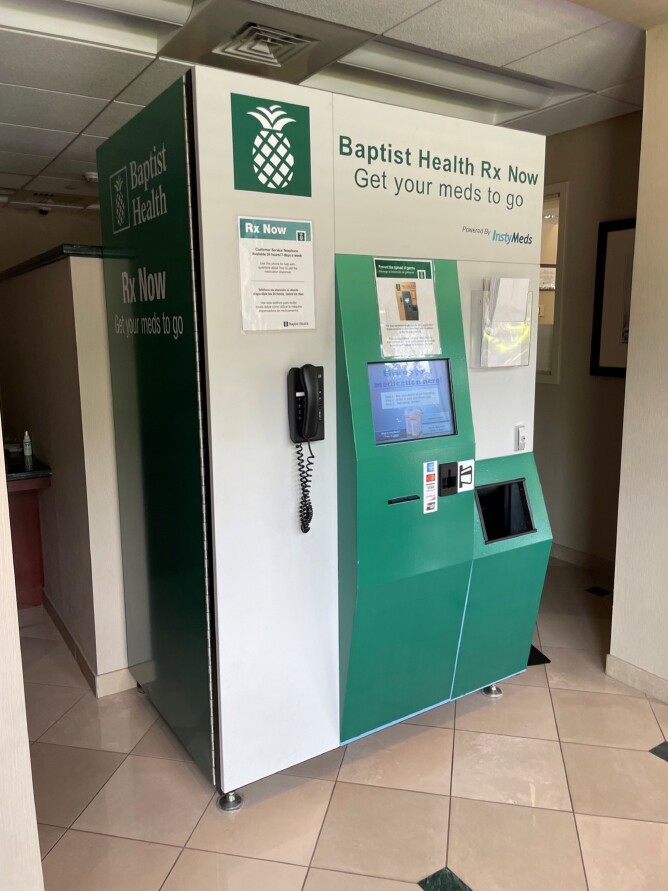

Example of an automated pharmacy dispensing machine found in UCCs throughout the healthcare system. Each machine contains frequently prescribed outpatient regimens for antibiotics, analgesics, antihistamines, and more. In cases where a patient is prescribed a stocked regimen, the machine is able to safely and conveniently dispense the medication prior to the patient leaving the UCC. Literature has shown automated pharmacy dispensing machines can promote patient medication adherence.

**Methods:**

The Cerner Discern Reporting Portal was used to generate FQ electronic prescription (eRx) data during 2019 (pre-intervention) and 2022 (post-intervention) to assess any potential sustained impact of the targeted interventions from 2020. The primary endpoint was a 10% reduction in total FQ eRx count, total FQ days of therapy (DOT), and median FQ DOT per monthly UCC patient volume compared to the pre-intervention period. UCC monthly patient volume was used to standardize days of therapy comparisons among the pre-intervention and post-intervention periods. Total calendar-year FQ DOT was assessed by summing up monthly values. The secondary endpoint was a median monthly FQ eRx count of less than 550 in the post-intervention period. Continuous data were analyzed using an unpaired t-test. Institutional Review Board approval was granted.

**Results:**

Total UCC annual patient volume was 337,941 in 2019 and 285,865 in 2022. Total FQ eRx count decreased from 7,214 in 2019 to 3,883 in 2022 (46% reduction, p=< 0.0001). The total FQ DOT in the calendar year decreased from 58,761 days in the pre-intervention period to 29,578 days in the post-intervention period (50% reduction, p=< 0.0001). Median monthly DOT per UCC patient volume decreased from 176 days per UCC monthly volume in 2019 to 106 days per UCC monthly volume in 2022 (40% reduction, p=< 0.0001). The median monthly FQ eRx count in 2022 was 318 which was substantially below our target of 550 and the median count of 602 in 2019.

**Figure:**
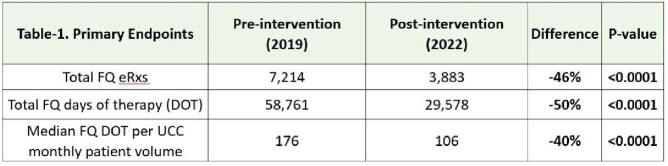

**Figure:**
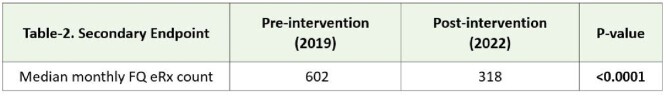

**Figure:**
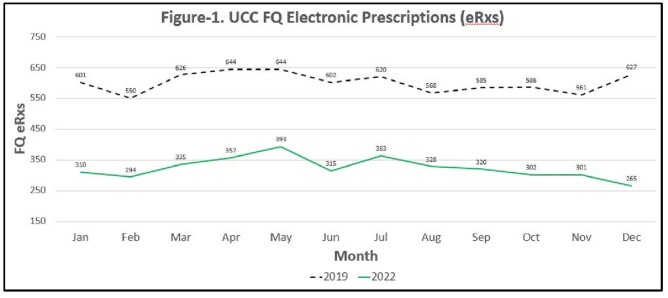

**Conclusion:**

Coordinated antimicrobial stewardship efforts in urgent care centers to reduce outpatient FQ consumption had a sustained impact following intervention implementation.

**Disclosures:**

**Timothy P. Gauthier, PharmD, BCPS, BCIDP**, American Pharmacist Association: Honoraria|Belmont University CME Office: Honoraria|Compact: Honoraria|European Society for Clinical Microbiology and Infectious Diseases: Honoraria|Ferring Pharma: Advisor/Consultant|Firstline Mobile Health: Advisor/Consultant|Florida Society of Health-System Pharmacists: Honoraria|Gilead Biosciences: Advisor/Consultant|GlaxoSmithKline Inc.: Advisor/Consultant|Learn Antibiotics and IDstewardship.com: Owner of all published content|Learn Antibiotics and IDstewardship.com: Owner|Learn Antibiotics and IDstewardship.com: Ownership Interest|Melinta Therapuetics: Advisor/Consultant|Pattern Bioscience: Advisor/Consultant|Pfizer, Inc.: Advisor/Consultant

